# Factors Related to Textbook Outcome in Laparoscopic Liver Resections: a Single Western Centre Analysis

**DOI:** 10.1007/s11605-022-05413-x

**Published:** 2022-08-12

**Authors:** Andrea Ruzzenente, Edoardo Poletto, Simone Conci, Tommaso Campagnaro, Bernardo Dalla Valle, Mario De Bellis, Alfredo Guglielmi

**Affiliations:** Department of Surgery, Dentistry, Gynaecology and Paediatrics, Division of General and Hepato-Biliary Surgery, University of Verona, P. le L.A. Scuro, 37134 Verona, Italy

**Keywords:** Laparoscopy, Liver surgery, Composite measure, Quality of care, Textbook outcome

## Abstract

**Introduction:**

The selection of the most informative quality of care indicator for laparoscopic liver surgery (LLS) is still debated; among those proposed, textbook outcome (TO) seems to provide a compositive measure of the outcomes of surgery.

The aim of this study was to investigate the factors related with the TO in a cohort of patients who underwent LLS.

**Methods:**

Patients who underwent LLS from 2014 to 2021 were included. TO for LLS (TOLLS) was defined as: R0 resection, absence of intraoperative incidents, severe complications, reintervention, 30-day readmission and in-hospital mortality. When also considering no prolonged length of hospital stay (LOS), the outcome was called TOLLS+.

**Results:**

Four hundred twenty-one patients were included; TOLLS was achieved in 80.5%, TOLLS+ in 60.8% cases. R0 resection was obtained in 90.2% cases, intraoperative incidents occurred in 7.8%, severe complications in 5.0%, reintervention in 0.7%, readmission in 1.4% and in-hospital mortality in 0.2%. 32.5% of patients showed prolonged LOS. After univariate and multivariate analysis, factors influencing TOLLS were age (OR 0.967; *p=*0.003), concomitant surgery (OR 0.380; *p=*0.003), operative time (OR 0.996; *p=*0.008) and blood loss (OR 0.241; *p<*0.001); factors influencing TOLLS+ were ASA-score (OR 0.533*; p=*0.008), tumour histology (OR 0.421; *p=*0.021), concomitant surgery (OR 0.293*; p<*0.001), operative time (OR 0.997; *p=*0.016) and blood loss (OR 0.361; *p=*0.003).

**Conclusions:**

TOLLS can be achieved in most patients undergoing LLR, and it seems to be influenced mostly by surgery-related factors; conversely, TOLLS+ is achieved less frequently and seems to be influenced also by patient- and tumour-related factors.

**Supplementary Information:**

The online version contains supplementary material available at 10.1007/s11605-022-05413-x.

## Background

In the last decades, laparoscopic liver surgery (LLS) has proven to be feasible and safe for the treatment of both benign and malignant liver diseases, showing benefits when compared with open surgery, especially in terms of post-operative morbidity and length of hospital stay.^1^

Alongside the worldwide spread of LLS, surgical quality assessment is becoming crucial; although surgical outcomes have been used as a tool for assessing quality, they typically do not reflect the multidimensionality of the surgical process. Moreover, the reliability of single-risk-adjusted outcome measure reported to be low for differentiating hospital performance.^2,3^ Given these reasons, the identification of the most informative quality of care indicator is still a matter of debate in literature.

Composite outcomes have been proposed to avoid these limitations, combining multiple outcomes into a single summary measure.^3^ The most known and used combined measure is called textbook outcome (TO), an all-or-none combined outcome tool that includes peri-operative outcomes indexes of an optimal peri-operative care. While TO was evaluated in many surgical areas and disciplines,^4–6^ a proper definition and evaluation for LLS is still lacking; furthermore, analysis of factors influencing TO is still controversial.

In a recently published, multicentric study, Görcec et al. tried to give a definition of textbook outcome for laparoscopic liver surgery (TOLLS) based on an internationally conducted survey involving members of the European-African and International Hepato-Pancreato-Biliary Association and identified the most important influencing factors.^7^

The aims of this study are to evaluate and validate the so-defined TOLLS on a western tertiary HPB referral centre case-series, and to analyse the factors related with its achievement.

## Materials and Methods

### Data Source and Study Population

Patient data were obtained from a prospectively maintained, anonymized database of all the patients undergoing LLS at General and Hepatobiliary Surgery Division of University of Verona, Italy. All patients who underwent surgery between January 2014 and June 2021 were considered for the study. Inclusion criteria were age ≥ 18 years, at least one laparoscopic liver resection performed, 90-day follow-up and the availability of data regarding intra-operative events, post-operative complications, length of hospital stay, post-operative readmission or mortality and state of the resection margins. Patients undergoing cyst fenestration or tumour ablation were excluded; all patients missing one or more data needed to evaluate the TO were excluded. This study was reviewed and approved by the Ethics committee of our institution.

### Data Collection

Demographic and clinical data analysed included were gender, age, body mass index (BMI), American Society of Anaesthesiologists (ASA) class, Charlson comorbidity score (CCs), liver disease, the presence of clinical portal vein hypertension (assessed by spleen diameter, presence of gastro-oesophageal varices or platelets count ≤ 100,000), platelets count, pathological diagnosis of the liver tumour, number of tumours, size of the main lesion and its proximity to major vessels. Moreover, we considered previous abdominal non-hepatic or hepatic surgery and neo-adjuvant chemotherapy, concomitant surgical procedures (small or large bowel resection, hepatic hilar and local lymphadenectomy, main bile duct resections and biliary-jejunal reconstruction). Minor surgical procedures as cholecystectomy or ventral/inguinal hernia repair were not considered. The types of resections were categorised (minor vs technically and anatomically major resections) according to the Brisbane nomenclature and the Southampton guidelines for laparoscopic liver surgery statements;^1,8^ in particular, while anatomically major resections were defined as resections of three or more Couinaud segments, whereas technically major resections as anatomical resections of one or two liver “difficult segments” (I, IVa, VII and VIII). The Iwate difficulty scoring systems (DSS) for LLS was also calculated in all cases.^9^ The following intra-operative aspects were considered: operative time, hilar clamping, blood losses and peri-operative (within 24 h) blood transfusions. Finally, length of hospital stay (LOS) was considered, divided in categories based on the extent of resection performed (minor and technically/anatomically major); prolonged hospital stay was defined as hospital stay equal or longer than the 75th percentile for every category.

### Textbook Outcome

**Fig. 1:**
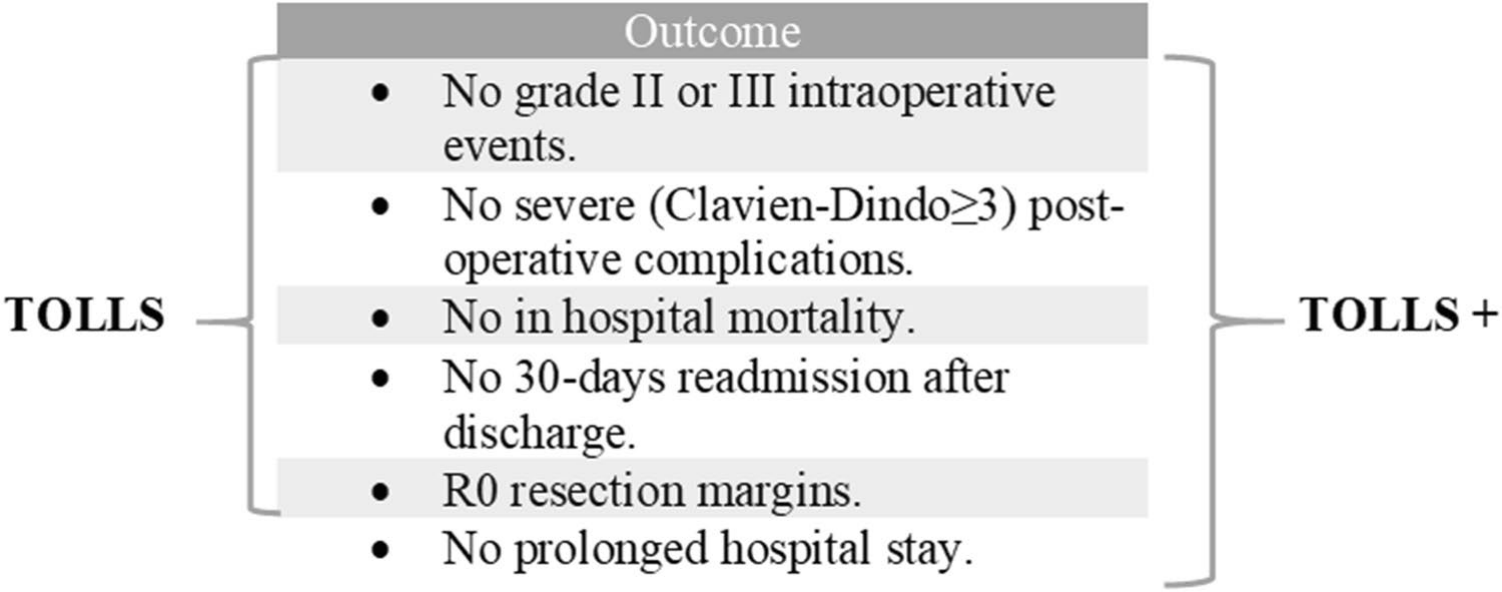
Definition of TOLLS and TOLLS+

In a recently published multicentric study, Görcec et al. proposed a definition of textbook outcome for laparoscopic liver surgery (TOLLS):^7^ the authors developed a survey including individual surgical outcomes and submitted to the European-African and International Hepato-Pancreato-Biliary Association (E-AHPBA, I-HPBA) members. Parameters included in the definition were the following: absence of grade II or III intra-operative events according to the Oslo classification; this is a classification that divides peri-operative events into three classes: I, incidents managed without changing the operative approach and without further consequences for the patient; II, incidents with further consequences for the patients (i.e. excessive blood losses, and for endoscopic surgery, anything requiring unplanned conversion) and III, incident leading to significant consequences for the patient (i.e. intra-operative death);^10^ no severe post-operative complications, classified according to the Clavien-Dindo classification^11^ as grade III or higher; no readmission within 30 days, no in-hospital mortality and R0 resection margins (defined as tumour-free margin of 1 mm or more). In the present study, it was applied this definition of TOLLS. Moreover, according to Görcec et al., an extended definition of TOLLS was created also considering the length of hospital stay (LOS); this enriched definition of TOLLS has been named as TOLLS+ (Figure 1).

### Statistical Analysis

Categorical variables were presented as frequency, while continuous variables were expressed as median and interquartile range (IQR). To investigate possible association among patients, tumour and operative characteristics and TOLLS both a univariate and a multivariate logistic regression analysis were performed. Factors that resulted significant after univariate analysis were considered for multivariate analysis. The same analysis was performed for TOLLS+. A *p* value of <0.05 was considered as significant. All statistical analyses were performed using SPSS (IBM Corp. Released 2012. IBM SPSS Statistics for Windows, Version 21.0. Armonk, NY: IBM Corp).

## Results

### Baseline Characteristics

A total of 421 patients who underwent LLS were included in the study population (Table 1). Most patients were male (*n*=251, 59.6%); median age was 66 years (IQR 55–74), and median BMI was 25.6 (IQR 23.4–27.9). Most patients had an ASA score of 1 or 2 (*n*=266, 63.2%), and median Charlson comorbidity score (CCs) was 3 (IQR 2–5); 153 patients showed liver cirrhosis (36.8%); the remaining had a healthy liver, while 76 patients showed signs of portal hypertension (18.1%). The median value for platelets count was 200 (10^3/mm^3^) (IQR 142–250). At final pathology, 74 patients (17.6%) showed a benign disease and 347 patients showed malignant tumours; in particular, non-colorectal liver metastasis (NCRLM) in 33 (7.8%), colorectal liver metastasis (CRLM) in 77 (18.3%), hepatocellular carcinoma (HCC) in 180 (42.8%) and biliary tract cancers (gallbladder carcinoma, intrahepatic and perihilar cholangiocarcinoma) in 57 (13.6%). Most patients presented a single lesion (*n*=295, 70.1%), while 126 patients showed multiple lesions; the tumour size was less than 3 cm in 43.9% (*n*=185), between 3 and 5 cm in 29.2% (*n*=123) and more than 5 cm in 26.8% (*n*=26.8%); location was near to main vessels (distance smaller than 2 cm from main portal branches and the hepatic veins) in 236 (56.1%). Of the 421 patients involved in this study, 240 (57%) had previous abdominal surgery, specifically 30 (7.1%) had previous liver surgery. According to the Iwate DSS, after excluding 4.8% of the patients in which it was not computable, 93 (22.1%) patients were in the low and 166 (39.3%) in the intermediate difficulty class, whereas 11 (26.1%) and 32 (7.6%) were in advanced and expert difficulty classes (Table 1). Most patients underwent minor liver resection (*n*=324, 77%), while 97 underwent technically or anatomically major resection (23%); a concomitant surgical procedure was performed in 72 (17.1%).

**Table 1:** Baseline features of MILS patients (*N*=421)

Variables	
Gender	
Male	251 (59.6%)
Female	170 (40.4%)
Age (years)	66 (55–74)
BMI	25.6 (23.4–27.9)
ASA score	
1–2	266 (63.2%)
3–4	155 (36.8%)
CCs	3 (2–5)
Liver histology	
Healthy	268 (63.7%)
Cirrhosis	153 (36.3%)
Portal vein hypertension*	
No	345 (81.9%)
Yes	76 (18.1%)
Platelets (10^3/mm^3^)	200 (142–250)
Disease	
Benign	74 (17.6%)
Malignant	347 (82.4%)
* HCC*	*180 (42.8%)*
* CRLM*	*77 (18.3%)*
* NCRLM*	*33 (7.8%)*
* CCC*	*57 (13.6%)*
No. of tumours	
Single	295 (70.1%)
Multiple	126 (29.9%)
Proximity to main vessels	
<2 cm	185 (43.9%)
≥2 cm	236 (56.1%)
Dimension of tumour (cm)	
< 3	185 (43.9%)
3–5	123 (29.2%)
> 5	113 (26.8%)
Previous abdominal surgery	240 (57%)
Previous liver surgery	30 (7.1%)
Neo-adjuvant chemotherapy	52 (12.4%)
Extent of resection	
Minor	324 (77%)
Technically or anatomically major	97 (23%)
Concomitant surgery	
No	349 (82.9%)
Yes	72 (17.1%)
Concomitant surgery description	
Bowel resections	19 (26.4%)
Hilar lymphadenectomy	39 (54.1%)
MBD resection	6(8.3%)
Other	8(11.2%)
Iwate DSS	
Low	93 (22.1%)
Intermediate	166 (39.4%)
Advanced	110 (26.1%)
Expert	32 (7.6%)
NC	20 (4.8%)
Operative time (min)	255 (180–330)
Hilar clamping	
No	143 (34%)
Yes	278 (66%)
Blood losses	
<500 ml	361 (85.7%9
≥500 ml	60 (14.3%)
Peri-operative blood transfusions	
No	393 (93.3%)
Yes	28 (6.7%)
Intra-operative events**	
No events	369 (87.6%)
I	19 (4.5%)
II	33 (7.8%)
III	0 (0%)
Complications	
No	306 (72.7%)
Clavien-Dindo 1–2	94 (22.3%)
Clavien-Dindo ≥3	21 (5%)
Length of hospital stay (days)	
Minor resections	5 (4–6)
Technically/anatomically major resections	6 (5–8)
Prolonged LOS	137 (32.5%)
In-hospital mortality	1 (0.2%)
30-day readmission rate	6 (1.4%)
30-day re-intervention rate	3 (0.7%)
Radicality of the resection	
R0	380 (90.3%)
R1	41 (9.8%)
TOLLS	
No	82 (19.5%)
Yes	339 (80.5%)
TOLLS+	
No	165 (39.2%)
Yes	256 (60.8%)

Signifcant *p* values (*p* < 0.05) are reported in italics

^*^Portal vein hypertension assessed by platelet count, spleen dimension, presence of oesophageal varices or collateral circulations

^**^Classified according to Modified Oslo Classification

*CCs*, Charlson comorbidity score; *NC*, not computable; *HCC*, hepatocellular carcinoma; *CRLM*, colon-rectal liver metastasis; *NCRLM*, non-colon-rectal liver metastasis; *CCC*, cholangio-cellular carcinoma; *DSS*, difficulty scoring system; *HS*, hospital stay; *TOLLS*, textbook outcome for laparoscopic liver surgery; *TOLLS*+, TOLLS + prolonged hospital stay

Median operative time was 255 (IQR 180–330), hilar clamping was applied in 278 (66%) and blood losses greater than 500 ml were registered in the 14.3% (*n*=60), while peri-operative transfusions were required in 28 (6.7%).

### Textbook Outcome

**Fig. 2:**
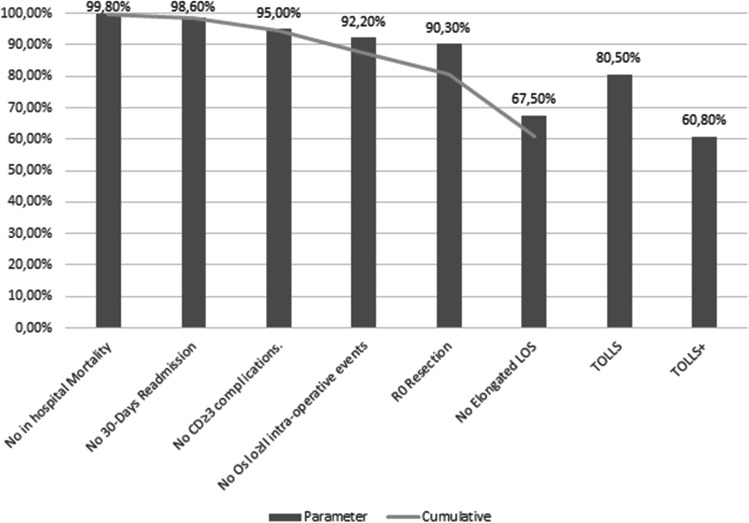
Single and cumulative frequencies of the surgical outcomes involved in calculation of TOLLS and TOLLS+

The frequencies of every single surgical outcome included in TOLLS and TOLLS+ were absence of in hospital mortality in 99.8%, no 30-day readmission in 98.6%, no severe (CD≥3) complications in 95%, no grade 2–3 intra-operative events in 92.2% and R0 resection in 90.3%. Overall, TOLLS was achieved in 80.5% of patients. When considering prolonged length of hospital stay (LOS) in calculating TOLLS+, no prolonged LOS was achieved in 67.5%; consequently, TOLLS+ was achieved in 60.8% (Fig. 2).

### Factors Associated with TOLLS

Results of univariate analysis for TOLLS are shown in Table 2. At univariate analysis, factors associated with non-achievement of TOLLS with a statistically significant relationship were age (odds ratio [OR] 0.964 [95% CI, 0.945–0.984]; *p=*0.001), technically/anatomically major resection (OR 0.427 [95% CI, 0.253–0.720]; *p=*0.001), concomitant surgery (OR 0.245 [95% CI, 0.141–0.426]; *p<*0.001), operative time (OR 0.993 [95% CI, 0.991–0.996]; *p<*0.001), hilar clamping (OR 0.566 [95% CI, 0.327–0.982]; *p=*0.043), blood losses (OR 0.184 [95% CI, 0.103–0.330]; *p<*0.001) and blood transfusions (OR 0.178 [95% CI, 0.081–0.392]; *p<*0.001); moreover, malignant histology was associated with a non-achievement of TOLLS (OR 0.254 [95% CI, 0.099–0.652]; *p=*0.004), and in particular histological diagnosis of cholangiocarcinoma (OR 0.093 [95% CI, 0.033–0.264]; *p<*0.001) or CRLM (OR 0.207 [95% CI, 0.073–0.585]; *p=*0.003). Finally, increased difficulty according to Iwate DSS showed statistically significant relationship with TOLLS, higher technical difficulty of surgery increases the risk not to achieve TOLLS and OR for expert class of difficulty was 0.132 (95% CI, 0.044–0.397, *p=*0.004).

**Table 2:** Univariate analysis for TOLLS and TOLLS+

	TOLLS		TOLLS+	
Variable	OR (95% CI)	*p* value	OR (95% CI)	*p* value
Sex				
Female	Ref		Ref	
Male	0.719 (0.434–1.192)	0.20	1.264 (0.846–1.889)	0.25
Age	0.964 (0.945–0.984)	*0.001*	0.964 (0.949–0.980)	*0.001*
BMI	1.012 (0.953–1.075)	0.69	0.998 (0.951–1.047)	0.93
ASA				
1–2	Ref		Ref	
3–4	0.691 (0.423–1.128)	0.14	0.422 (0.280–0.634)	*<0.001*
CCs	0.932 (0.844–1.030)	0.17	0.858 (0.789–0.934)	*0.001*
Liver histology				
Healthy	Ref		Ref	
Cirrhosis	1.383 (0.823–2.324)	0.22	0.878 (0.585–1.317)	0.53
Portal hypert.				
No	Ref		Ref	
Yes	1.087 (0.575–2.057)	0.80	0.664 (0.402–1.094)	0.108
Platelets	0.999 (0.997–1.002)	0.48	1.001 (0.999–1.003)	0.36
Lesion histology				
Benign	Ref		Ref	
Malignant	0.254 (0.099–0.652)	*0.004*	0.219 (0.111–0.430)	*0.001*
Disease				
HCC	0.393 (0.146–1.062)	0.07	0.256 (0.126–0.519)	*<0.001*
CRLM	0.207 (0.073–0.585)	*0.003*	0.210 (0.096–0.458)	*<0.001*
NCRLM	0.525 (0.132–2.098)	0.36	0.349 (0.133–0.918)	*0.033*
CCC	0.093 (0.033–0.264)	*>0.001*	0.110 (0.048–0.253)	*<0.001*
Benign	Ref		Ref	
N_O_. of tumours				
Single	Ref		Ref	
Multiple	1.207 (0.703–2.072)	0.49	1.018 (0.664–1.562)	0.93
Dimension of tumour (cm)				
<3	Ref		Ref	
3–5	0.737 (0.419–1.296)	0.29	0.941 (0.590–1.500)	0.79
>5	0.951 (0.519–1.742)	0.87	0.941 (0.583–1.520)	0.80
Prox. to vessels				
No	Ref		Ref	
Yes	0.695 (0.428–1.127)	0.14	0.654 (0.441–0.971)	*0.035*
Prev. abdominal surgery				
No	Ref		Ref	
Yes	0.816 (0.499–1.336)	0.38	0.887 (0.597–1.318)	0.55
Prev. liver surgery				
No	Ref		Ref	
Yes	0.965 (0.381–2.444)	0.94	2.228 (0.934–5.317)	0.07
Neo-adjuvant chemotherapy				
No	Ref		Ref	
Yes	0.690 (0.350–1.362)	0.28	0.660 (0.368–1.183)	0.16
Extent of resection				
Minor	Ref		Ref	
Technically/anatomically major	0.427 (0.253–0.720)	*0.001*	0.718 (0.454–1.136)	0.16
Concomitant surgery				
No	Ref		Ref	
Yes	0.245 (0.141–0.426)	*<0.001*	0.200 (0.115–0.348)	*<0.001*
Iwate DSS				
Low	Ref		Ref	
Intermediate	0.389 (0.153–0.986)	*0.047*	0.802 (0.465–1.385)	0.43
Advanced	0.168 (0.067–0.423)	*<0.001*	0.480 (0.269–0.858)	*0.013*
Expert	0.132 (0.044–0.397)	*<0.001*	0.431 (0.189–0.980)	*0.04*
Operative time (min)	0.993 (0.991–0.996)	*<0.001*	0.994 (0.992–0.996)	*<0.001*
Hilar clamping				
No	Ref		Ref	
Yes	0.566 (0.327–0.982)	*0.043*	0.834 (0.550–1.265)	0.39
Blood losses				
<500 ml	Ref		Ref	
≥500 ml	0.184 (0.103–0.330)	*<0.001*	0.242 (0.135–0.436)	*<0.001*
Blood transfusions				
No	Ref		Ref	
Yes	0.178 (0.081–0.392)	*<0.001*	0.156 (0.062–0.394)	*<0.001*

Signifcant *p* values (*p* < 0.05) are reported in italics

Multivariate analysis showed that factors related with non-achievement of TOLLS were age (OR 0.967 [95% CI, 0.945–0.989]; *p=*0.003), concomitant surgery (OR 0.380 [95% CI, 0.199–0.724]; *p=*0.003), operative time (OR 0.996 [95% CI, 0.994–0.999]; *p=*0.008) and blood losses (OR 0.241 [95% CI, 0.124–0.466]; *p<*0.001). (Table 3)

**Table 3:** Multivariate analysis for TOLLS

	TOLLS	
Variable	OR (95% CI)	*p* value
Age	0.967 (0.945–0.989)	*0.003*
Lesion histology		0.68
Benign	Ref	
Malignant	0.796 (0.264–2.399)	
Extent of resection		
Minor	Ref	
Technically/anatomically major	0.769 (0.405–1.446)	0.41
Concomitant surgery		
No	Ref	
Yes	0.380 (0.199–0.724)	*0.003*
Operative time (min)	0.996 (0.994–0.999)	*0.008*
Hilar clamping		
No	Ref	
Yes	0.852 (0.454–1.598)	0.62
Blood losses		
<500 ml	Ref	
≥500 ml	0.241 (0.124–0.466)	*<0.001*
Blood transfusions		
No	Ref	
Yes	0.6 (0.236–1.583)	0.31

Signifcant *p* values (*p* < 0.05) are reported in italics

### Factors Associate with TOLLS+

The extended definition of TOLLS, including also prolonged hospital stay (TOLLS+) was also investigated through both multivariate and univariate analysis. At univariate analysis, age (OR 0.964 [95% CI, 0.949–0.980]; *p=*0.001), concomitant surgery (OR 0.200 [95% CI, 0.115–0.348]; *p<*0.001), operative time (OR 0.994 [95% CI, 0.992–0.996]; *p<*0.001), blood losses (OR 0.242 [95% CI, 0.135–0.436]; *p<*0.001) and transfusions (OR 0.156 [95% CI, 0.062–0.394]; *p<*0.001) had negative impact on the achievement of TOLLS+ (Table 2). Moreover, malignant histology (OR 0.219 [95% CI, 0.111–0.430]; *p=*0.001), higher difficulty classes calculated with Iwate DSS (OR 0.431 [95% CI, 0.189–0.980]; *p=*0.04 for expert difficulty class), ASA score 3/4 (OR 0.422 [95% CI, 0.280–0.634]; *p<*0.001) and CCs (OR 0.858 [95% CI, 0.789–0.934]; *p=*0.001) were associated with not achieving TOLLS+.

At multivariate analysis, factors related with TOLLS+ were ASA score (OR 0.533 [95% CI, 0.336–0.847]; *p=*0.008), malignant histology (OR 0.421 [95% CI, 0.202–0.879]; *p=*0.021), concomitant surgery (OR 0.293 [95% CI, 0.153–0.548]; *p<*0.001), operative time (OR 0.997 [95% CI, 0.995–0.999]; *p=*0.016) and blood losses (OR 0.361 [95% CI, 0.186–0.702]; *p=*0.003) (Table 4). Age, while reaching significance after univariate analysis, was not included in multivariate analysis since increasing age was associated with increasing ASA score, thus invalidating the analysis.

**Table 4:** Multivariate analysis for TOLLS+

	TOLLS+	
Variable	OR (95% CI)	*p* value
ASA		
1–2	Ref	
3–4	0.533 (0.336–0.847)	*0.008*
CCs	0.947 (0.845–1.061)	0.35
Lesion histology		
Benign	Ref	
Malignant	0.421 (0.202–0.879)	*0.021*
Prox. to vessels		
No	Ref	
Yes	0.703 (0.444–1.113)	0.13
Concomitant surgery		
No	Ref	
Yes	0.293 (0.153–0.548)	*<0.001*
Operative time (min)	0.997 (0.995–0.999)	*0.016*
Blood losses		
<500 ml	Ref	
≥500 ml	0.361 (0.186–0.702)	*0.003*
Blood transfusions		
No	Ref	
Yes	0.436 (0.149–1.273)	0.13

Signifcant *p* values (*p* < 0.05) are reported in italics

A subset analysis comparing major (technically and anatomically) and minor resections has been carried out and the results can be found in the supplementary material (Supplementary Tables 1, 2, 3 and 4).

## Discussion

Quality assessment in surgery has become of paramount importance, especially in a novel and improvable field such as LLS. Traditionally, quality assessment relied on the analysis and comparison of a single simple surgical outcome, such as mortality, morbidity, hospital stay or readmission;^12–14^ many authors underline the inadequacy of the single-outcome approach to describe the complexity and multidimensionality of the surgical process.^2,15^

The use of composite outcomes has been suggested to properly analyse and compare performances. Among these, textbook outcome (TO) has been indicated as the best tool for evaluating surgical outcomes. Moreover, TO, an all-or-none composite outcome tool, combines multiple single binary outcomes and can be achieved only when all outcomes are achieved. This is a more comprehensive patient-focused evaluation of the surgical performance because a favourable outcome is obtained only when all the items are satisfied. The TO has been defined and validated for many surgical procedures, including liver surgery, but a specific TO definition for LLS still lacking. Görcec et al.^7^ performed a survey among experts in this branch of surgery, in order to develop a new definition of TO that should be better suited for LLS. In our study, we applied this definition of TO for LLS (TOLLS) in a cohort of patients undergoing LLS in a single, tertiary referral HPB Western centre; moreover, we aimed to validate its performance and identify factors influencing TOLLS achievement.

In our case series, 80.5% of patients reached TO, a value which is in line with the recent published data, Görcec et al.^7^ in a multicentric study reported a rate ranging from 60.6 to 90.9% among different hospitals. Other authors found similar values: Merath et al. reported in a multicentric study a rate ranging from 16.6 to 78.7%,^6^ while Tsilimigras et al. and Mehta reported to have reached TO in 62.0% and 62.3%, respectively.^16,17^

When compared with open surgery, LLS seems to be more frequently associated with TO; Görcec et al. reported a rate of TO achievement of 70.7% for LLS and 66.8% of the patients undergoing open resection; this difference was statistically significant;^7^ similarly, Brustia et al., in a multicentric study on 855 patients undergoing liver resection for intra-hepatic cholangiocarcinoma, found that TO was achieved in 43.3% of LLS patients and 30.3% of open surgery patients; this difference was not statistically significative.^18^

In our results, radicality of the resection was the factor with the most negative impact on TOLLS achievement; the observed R0 resection rate of 90.3% is similar to other reports in literature. Görcec et al.^7^ and Tsilimigras et al.^16^ reported an R0 rate of 87.4% and 89%, respectively, and they confirmed that it was the most limiting factor in achieving TOLLS also in their experience. Conversely, in other case series, severe post-operative complications were the most negatively influencing TO for open or laparoscopic liver surgery, excluding LOS; specifically, Tsilimigras et al. reported a frequency of 14% of severe complications, while Azoulay et al. reported a frequency of 27%.^16,19^

Many factors have been found to influence the chance to achieve TO in published reports; in gastrointestinal surgery, three groups of factors have been reported: patient-related factors (age, comorbidities), tumour-related factors (stage of the tumour, dimension) and surgery-related factors (such as type of surgery).^4,5^

When considering TO for liver surgery, Tsilimigras et al. found similar results, identifying that patient-related (age, ASA score≥3), tumour-related (histology, vascular invasion) and surgery-related (type of liver resection) factors have a statistically significant relation with TO achievement. ^16^ These results were partially confirmed for LLS; TOLLS achievement seems to be less likely in advanced age, higher ASA class, previous abdominal surgery, malignant histology, tumour size and type of liver resection.^7^ In our report, we found that factors associated with a reduced chance of TOLLS achievement were mostly associated with the complexity of surgical procedure (concomitant surgery, blood losses and operative time). Not surprisingly, both TOLLS and TOLLS+ were negatively influenced by higher risk classes of Iwate DSS and the need of concomitant surgery.

In our study, when LOS is included in the definition of TOLLS, it becomes the outcome with the higher negative impact:^7,16,20^ TOLLS plus LOS (TOLLS+) was achieved in 60.8%, since prolonged LOS had a rate of 32.5%. This is comparable to other rates reported in literature: for example, in a recent multicentric study on liver resection for malignancies, a rate of prolonged LOS varied from 33.3 to 74.3%.^21^ Prolonged LOS has been deemed surrogate of many surgical outcomes such as post-operative complications, and that is why in many definitions of TO for complex surgeries, like colorectal or hepato-pancreato-biliary surgery, prolonged LOS was included.^4–7,16,20^ Many authors, on the other hand, believe that LOS is too susceptible to cultural or socio-economic factors with high variation rate among different countries and centres, implying it should not be included in the TO definition; Merath et al.,^21^ for example, that there are significant differences in prolonged LOS incidence between Eastern and Western hospital, suggesting that this may have cultural and organisational reasons, but maybe also reasons linked to economic interests and founding mechanisms. Given this dichotomy, we decided to test both definitions of TOLLS, with and without prolonged LOS, to highlight difference in factors limiting their achievement. Our results seem to suggest that when LOS is included in TOLLS definition, patient- and tumour-related factors have a somewhat higher impact in determining TO achievement, even if surgical complexity factors remain the ones with the bigger impact. Therefore, it is possible that TOLLS + could be more useful for proper and more accurate patient pre-selection and risk classification than TOLLS alone, even if this means achieving it in a lesser number of cases.

The results of this study should be evaluated in light of some limitations: firstly, its retrospective nature, although data were collected in a prospectively maintained database and in a short time of observation; secondly, we applied the definitions of TOLLS proposed by Görcec et al. that^7^ that have not still been externally validated; finally, the mono-centricity nature of our study, whereas standardisation of treatment (no variations in patient selection criteria, pre- and post-operative management, only three senior surgeons performed all the laparoscopic liver resections), may increase the value of our analysis.

## Conclusions

Textbook outcome is a simple, patient-centred tool to evaluate surgical performance also for LLS. According to our results, TOLLS can be achieved in most patients undergoing LLS, and it seems to be influenced mostly by surgery-related factors; conversely, when also LOS is considered, TOLLS+ is achieved less frequently and seems to be influenced by both patient- and tumour-related factors.

## Supplementary Information

Below is the link to the electronic supplementary material.

Supplementary file1 (DOCX 29.7 KB)

## Data Availability

The database gathering all data used for this article is available for the Editor of this article for review.

## Abbreviations

LLS: Laparoscopic liver surgery
TO: Textbook outcome
TOLLS: Textbook outcome for laparoscopic liver surgery
BMI: Body mass index
ASA: American Society of Anaesthesiologists
CCs: Charlson comorbidity score
DSS: Difficulty scoring systems
IQR: Inter-quartile range
LOS: Length of hospital stay
