# Supplementary material for: Factors Related to Textbook Outcome in Laparoscopic Liver Resections: a Single Western Centre Analysis
Source: J Gastrointest Surg. 2022 Aug 12;26(11):2301–10. doi: 10.1007/s11605-022-05413-x (PMC9643260; doi:10.1007/s11605-022-05413-x)
Supplement: Supplementary file 1 — Supplementary file1 (DOCX 29.7 KB) [file 11605_2022_5413_MOESM1_ESM.docx]

**Supplementary Table 1. Subset analysis comparing anatomically and technically major vs minor resections in terms of TOLLS, TOLLS+ and the single items included in them. The comparison has been carried out with Chi-squared test.**

|  | **Extent of resection** | | **P value** |
| --- | --- | --- | --- |
|  | **Minor** | **Major** |  |
| **Radicality**  **R0**  **R1** | 296 (91.4%)  28 (8.6%) | 84 (86.6%)  13 (13.4%) | 0.16 |
| **Intra-operative events**  **Grade 0-I**  **Grade II-III** | 302 (93.2%)  22 (6.8%) | 86 (88.7%)  11 (11.3%) | 0.14 |
| **Severe complications**  **No**  **Yes** | 313 (96.6%)  11 (3.4%) | 87 (89.7%)  10 (10.3%) | *0.006* |
| **90-days mortality**  **No**  **Yes** | 324 (100%)  0 (0%) | 96 (99%)  1 (1%) | 0.067 |
| **90-days re-intervention**  **No**  **Yes** | 322 (99.4%)  2 (0.6%) | 96 (99%)  1 (1%) | 0.67 |
| **90-days re-admission**  **No**  **Yes** | 322 (99.4%)  2 (0.6%) | 93 (95.9%)  4 (4.1%) | *0.011* |
| **Prolonged LOS**  **No**  **Yes** | 216 (66.7%)  108 (33.3%) | 68 (70.1%)  29 (29.9%) | 0.53 |
| **TOLLS**  **No**  **Yes** | 52 (16%)  272 (84%) | 30 (30.9%)  67 (69.1%) | *0.001* |
| **TOLLS+**  **No**  **Yes** | 121 (37.3%)  203 (62.7%) | 44 (45.4%)  53 (54.6%) | 0.16 |

**Supplementary Table 2: Subset analysis comparing anatomically and technically major vs minor resections in terms of factors found out to influence the achievement of TOLLS and TOLLS+. The comparison has been carried out with Chi-squared test, for this analysis the variables “age” and “operative time” have been dichotomized.**

|  | **Extent of resection** | | **P value** |
| --- | --- | --- | --- |
|  | **Minor** | **Major** |  |
| **Age**  **<65 years**  **≥65 years** | 163 (50.3%)  161 (49.7%) | 38 (39.2%)  59 (60.8%) | 0.054 |
| **ASA score**  **1-2**  **3-4** | 208 (64.2%)  116 (35.8%) | 58 (59.8%)  39 (40.2%) | 0.43 |
| **Lesion Histology**  **Benign**  **Malignant** | 61 (18.8%)  263 (81.2%) | 13 (13.4%)  84 (86.6%) | 0.22 |
| **Concomitant surgery**  **No**  **Yes** | 278 (85.8%)  46 (14.2%) | 71 (73.2%)  26 (26.8%) | *0.004* |
| **Blood losses**  **<500 ml**  **≥500 ml** | 287 (88.6%)  37 (11.4%) | 74 (76.3%)  23 (23.7%) | *0.002* |
| **Operative time**  **<240 min**  **≥240 min** | 156 (48.1%)  158 (51.9%) | 6 (6.2%)  91 (93.8%) | *<0.001* |

**Supplementary Table 3: Univariate analysis for TOLLS and TOLLS+ in the subset of major resections.**

|  | TOLLS | | TOLLS+ | |
| --- | --- | --- | --- | --- |
| Variable | **OR (95% CI)** | ***P* value** | **OR (95% CI)** | ***P* value** |
| Sex  Male  Female | Ref  1.676 (0.693-4.056) | 0.25 | Ref  1.391 (0.621-3.117) | 0.42 |
| Age  <65 years  ≥65 years | Ref  1.051 (0.436-2.535) | 0.91 | Ref  0.805 (0.354-1.832) | 0.60 |
| BMI | 1.003 (0.892-1.127) | 0.96 | 1.013 (0.909-1.129) | 0.81 |
| ASA  1-2  3-4 | Ref  0.829 (0.346-1.986) | 0.67 | Ref  0.671 (0.296-1.518) | 0.34 |
| CCs | 0.956 (0.784-1.165) | 0.66 |  |  |
| Liver Histology  Healthy  Cirrhosis | Ref  0.587 (0.233-1.480) | 0.26 | Ref  0.941 (0.390-2.272) | 0.893 |
| Portal Hypert.  No  Yes | Ref  0.524 (0.130-2.109) | 0.36 | Ref  1.042 (0.262-4.141) | 0.95 |
| Platelets | 1.00 (0.996-1.004) | 0.99 | 0.999 (0.995-1.003) | 0.57 |
| Lesion Histology  Benign  Malignant | Ref  0.633 (0.161-2.490) | 0.51 | Ref  0.489 (0.140-1.712) | 0.26 |
| N° of Tumours  Single  Multiple | Ref  0.793 (0.294-2.140) | 0.65 | Ref  1.396 (0.538-3.623) | 0.49 |
| Dimension of Tumour  <3 cm  3-5 cm  >5 cm | Ref  1.257 (0.449-3.523)  1.714 (0.588-4.994) | 0.66  0.32 | Ref  1.754 (0.656-4.689)  1.754 (0.656-4.689) | 0.26  0.26 |
| Prox. to Vessels  No  Yes | Ref  1.567 (0.638-3.848) | 0.33 | Ref  1.595 (0.681-3.738) | 0.28 |
| Prev. Abdominal Surgery  No  Yes | Ref  0.788 (0.319-1.948) | 0.61 | Ref  1.127 (0.493-2.575) | 0.78 |
| Prev. Liver Surgery  No  Yes | Ref  0.524 (0.130-2.109) | 0.36 | Ref  1.042 (0.262-4.141) | 0.95 |
| Concomitant Surgery  No  Yes | Ref  0.315 (0.123-0.808) | *0.016* | Ref  0.114 (0.380-0.341) | *<0.001* |
| Operative Time (min)  <240 min  ≥240 min | Ref  0.998 (0.994-1.002) | *0.35* | Ref  0.994 (0.989-0.998) | *0.005* |
| Hilar Clamping  No  Yes | Ref  0.362 (0.112-1.171) | 0.90 | Ref  0.819 (0.322-2.082) | 0.67 |
| Blood Losses  <500 ml  ≥500 ml | Ref  0.295 (0.111-0.781) | *0.011* | Ref  0.203 (0.071-0.576) | *0.003* |
| Blood Transfusions  No  Yes | Ref  0.595 (0.190-1.860) | 0.37 | Ref  0.344 (0.108-1.098) | 0.072 |

**Supplementary Table 4: Univariate analysis for TOLLS and TOLLS+ in the subset of minor resections.**

|  | TOLLS | | TOLLS+ | |
| --- | --- | --- | --- | --- |
| Variable | **OR (95% CI)** | ***P* value** | **OR (95% CI)** | ***P* value** |
| Sex  Female  Male | Ref  1.701 (0.891-3.247) | 0.108 | Ref  1.255 (0.788-1.999) | 0.34 |
| Age  <65 years  ≥65 years | Ref  0.340 (0.178-0.648) | *0.001* | Ref  0.381 (0.239-0.607) | *<0.001* |
| BMI | 1.021 (0.949-1.098) | 0.579 | 0.992 (0.940-1.046) | 0.77 |
| ASA  1-2  3-4 | Ref  0.596 (0.327-1.087) | 0.09 | Ref  0.387 (0.242-0.620) | *<0.001* |
| CCs | 0.928 (0.824-1.046) | 0.22 | 0.830 (0.754-0.914) | *<0.001* |
| Liver Histology  Healthy  Cirrhosis | Ref  1.675 (0.877-3.199) | 0.12 | Ref  0.787 (0.497-1.248) | 0.31 |
| Portal Hypert.  No  Yes | Ref  1.113 (0.527-2.355) | 0.78 | Ref  0.579 (0.336-0.998) | *0.049* |
| Platelets | 0.999 (0.996-1.002) | 0.65 | 1.003 (1.000-1.005) | 0*.*058 |
| Lesion Histology  Benign  Malignant | Ref  0.114 (0.034-0.611) | *0.009* | Ref  0.169 (0.074-0.386) | *<0.001* |
| N° of Tumours  Single  Multiple | Ref  1.484 (0.754-2.918) | 0.25 | Ref  0.968 (0.597-1.569) | 0.89 |
| Dimension of Tumour  <3 cm  3-5 cm  >5 cm | Ref  0.836 (0.416-1.681)  0.933 (0.445-1.956) | 0.61  0.85 | Ref  0.866 (0.507-1.497)  0.964 (0.551-1.689) | 0.6  0.9 |
| Prox. to Vessels  No  Yes | Ref  0.636 (0.350-1.159) | 0.14 | Ref  0.505 (0.318-0.804) | *0.004* |
| Prev. Abdominal Surgery  No  Yes | Ref  0.975 (0.537-1.772) | 0.93 | Ref  1.295 (0.824-2.035) | 0.26 |
| Prev. Liver Surgery  No  Yes | Ref  1.157 (0.328-4.080) | 0.82 | Ref  1.985 (0.708-5.563) | 0.19 |
| Concomitant Surgery  No  Yes | Ref  0.279 (0.138-0.562) | *<0.001* | Ref  0.260 (0.135-0.500) | *<0.001* |
| Operative Time (min)  <240 min  ≥240 min | Ref  0.992 (0.989-0.995) | *<0.001* | Ref  0.993 (0.990-0.996) | *<0.001* |
| Hilar Clamping  No  Yes | Ref  0.586 (0.303-1.133) | 0.11 | Ref  0.731 (0.455-1.175) | 0.196 |
| Blood Losses  <500 ml  ≥500 ml | Ref  0.163 (0.078-0.341) | *<0.001* | Ref  0.209 (0.099-0.442) | *<0.001* |
| Blood Transfusions  No  Yes | Ref  0.094 (0.026-0.334) | *<0.001* | Ref  0.054 (0.007-0.431) | *0.006* |
